# Performance and statistical characteristics of ferronickel slag geopolymer concrete

**DOI:** 10.1038/s41598-025-21614-1

**Published:** 2025-10-28

**Authors:** Shanshan Wang, Qun Huang, Zihong Xu

**Affiliations:** 1 School of Management Engineering, Zhejiang Guansha Vocational and Technical University of Construction, Dongyang, 322100 China; 2 Wuzhou Engineering Consulting Corporation Limited, Hangzhou, 310000 China

**Keywords:** Ferro–Nickel slag, Geopolymer concrete, Strength, Slump, Statistical characteristic, Engineering, Materials science

## Abstract

This study investigates the feasibility of substituting river sand with ferronickel slag (FNS) in geopolymer concrete (GC) through comprehensive experiments on mixtures with varying water-to-binder ratios, aggregate-to-binder ratios, and FNS replacement levels. Key properties evaluated include workability (16–30 cm slump, classified as high-fluidity concrete), compressive strength, splitting tensile strength, microstructure, and compressive strength statistics. Results indicate that increasing FNS replacement reduces slump, while higher aggregate-to-binder ratios or lower water-to-binder ratios further decrease workability. FNS-GC exhibits significant early-age strength development, achieving 72% and 94% of standard 28-day compressive strength at 3 and 14 days, respectively, with peak compressive strength observed at 33% FNS content. Similarly, splitting tensile strength (3.2- 4.4 MPa) reaches 75% and 93% of its 28-day value by 3 and 14 days, peaking at 33% FNS replacement and showing positive correlation with compressive strength. Statistically, compressive strength displays considerable variability (coefficient of variation: 0.07–0.17), maximized at 33% FNS replacement. The strength distribution aligns with normal, log-normal, and Weibull functions, with the Weibull distribution providing the optimal fit for characterizing compressive strength behavior.

**Supplementary Information:**

The online version contains supplementary material available at 10.1038/s41598-025-21614-1.

## Introduction

Concrete is a composite material formed by cementitious binders that tightly bond aggregates, exhibiting high mechanical strength and superior durability. Compared to other construction materials, it offers distinct advantages in raw material accessibility- key components like sand and gravel are widely available- and cost competitiveness due to industrial-scale production of binders.

Ordinary Portland Cement (OPC), the most commonly used binder, however, involves an energy-intensive manufacturing process. OPC production accounts for approximately 5% of global CO_2_ emissions [1, 2], making it a significant contributor to anthropogenic carbon footprints. With the continuous growth of the global population and rapid urbanization, the demand for concrete in civil engineering is expected to increase significantly in the coming years. To address the depletion of natural resources and mitigate irreversible ecological damage caused by production demands, developing clean, efficient, and low-carbon construction materials has become a key direction for the sustainable development of civil engineering.

Incorporating recyclable materials as substitutes for traditional concrete components not only reduces environmental pollution but also lowers construction costs and facilitates the establishment of a circular material recycling system [3]. Geopolymer concrete, a special class of cementitious composites, represents a highly promising alternative to traditional concrete and an innovative material paradigm. Renowned for its significantly reduced CO_2_ footprint, it is often termed “green concrete” [4, 5]. Unlike calcium silicate hydrate in Portland cement systems, geopolymer cement based on aluminosilicate chemistry introduces a fundamental shift in both the chemical composition of building materials and their synthesis pathways [6]. There are currently many studies on geopolymer concrete. For example, Nassar et al. [7] used finely ground blast furnace slag as a precursor and synthesized powder activators using waste glass powder, rice husk ash, and industrial grade sodium hydroxide to investigate the durability of single component alkali activated concrete against acid and sulfate corrosion. Marathe et al. [8] evaluated the environmental performance of air cured alkali activated permeable concrete developed using agricultural by-products, including sugarcane bagasse ash, recycled concrete aggregates, waste casting sand, and finely ground blast furnace slag. By integrating agricultural and industrial waste streams into concrete composite production, this study advances the principles of resource-saving engineering and circular economy. Doping heavy metal ions into adhesives is an effective strategy to combat corrosion caused by microorganisms in wastewater systems. Li et al. [9] utilized incineration sludge ash containing trace heavy metals to provide bactericidal effects in alkaline active materials prepared from GGBS and waste glass powder. Then expose the obtained mixture to a real sewage environment for 24 months. Marathe et al. [10] investigated the feasibility of using alkali activated ground blast furnace slag and sugarcane bagasse ash to enhance the stability of red soil subgrade. The experimental results indicate that when sugarcane bagasse ash is added, the strength of the roadbed soil increases to a threshold of 10%. However, beyond this threshold, strength will decrease. A comprehensive cost exploration comparing traditional and improved pavement designs shows the economic benefits of using stabilization measures in rural pavement construction while maintaining sustainability.

The geopolymerization process requires alkaline activation, which fundamentally relies on two key components: precursors and alkaline activators. Traditionally used precursors include aluminosilicate materials such as calcined clays (e.g., metakaolin), fly ash (FA), and blast furnace slag [11]. Common activators- typically sodium or potassium-based- such as hydroxide solutions or sodium silicate, when combined with precursors, generate dense cementitious matrices with properties comparable to or even superior to Ordinary Portland Cement (OPC) [12, 13]. Currently, blast furnace slag (BFS) and FA, which are rich in aluminosilicate content, are the most widely used precursors in geopolymer systems [14–16]. FA, known for its slow reactivity and requirement for high-temperature curing, is often used in combination with ground blast furnace slag (GBFS) to achieve synergistic effects. This composite approach enhances the overall performance of alkali-activated materials and enables effective curing at ambient temperatures [17].

The production of alkali-activated binders based on GBFS/FA blends has gained significant traction due to their versatility in enabling customized binder formulations [18]. Research has identified the primary reaction products of the BFS/FA system as a mixture of C-A-S–H (calcium-alumino-silicate-hydrate) and N-A-S–H (sodium-alumino-silicate-hydrate) gels [19, 20]. The increased reactivity of the mixed precursors, coupled with the stable coexistence of C-A-S–H and N-A-S–H type gels, facilitates the formation of binder matrices that are denser and less porous compared to those derived from pure BFS or FA alone [21, 22]. Singh et al. [23] investigates the impact of various mix design parameters on Compressive Strength (CS) and fresh properties of SCGPC.

On the other hand, aggregates are indispensable components of concrete, accounting for 65% to 80% of its total volume. According to the 4.75 mm particle size boundary, aggregates are classified into coarse aggregates and fine aggregates [24]. The bonding of coarse and fine aggregates with cementitious materials forms a robust skeleton, which enhances concrete stability, mitigates shrinkage, and improves abrasion resistance.

NS is the most widely used fine aggregate in concrete preparation. However, the massive consumption of natural sand for concrete production has led to severe environmental degradation. Thus, developing industrial by-products as eco-friendly substitutes for natural sand holds significant promise for promoting sustainable construction practices.

Ferronickel slag (FNS), a by-product generated during ferronickel alloy smelting, primarily originates from blast furnace and electric furnace processes, resulting in blast furnace FNS and electric furnace FNS, respectively. FNS is formed when these by-products are cooled by water or air during smelting [25]. It is estimated that approximately 14 tons of FNS are generated per ton of nickel produced [24, 26]. Currently, the majority of FNS is disposed of through stockpiling, leading to both resource waste and potential environmental pollution risks. Over recent decades, FNS has undergone extensive research for its reuse potential, demonstrating applications as sandblasting materials, anti-slip pavement aggregates, admixtures in cement production, and inert additives or aggregates in concrete manufacturing [25, 27].

FNS has been proven to be an excellent raw material for geopolymer production. With proper mix design, it enables the production of geopolymers exhibiting significantly high strength and low water absorption [28]. Nguyen and Castel [29] developed a GGBFS-FNS geopolymer using FNS and ground granulated blast-furnace slag (GGBFS). This geopolymer exhibited high resistance to chloride diffusion, non-reactivity to alkali-silica reaction (ASR), and superior sulfate resistance. Overall, the GGBFS-FNS geopolymer demonstrates significant potential as an eco-friendly construction material, particularly for applications in aggressive environments.

When ground into powder, FNS can be used as a raw material for geopolymer production. Alternatively, when crushed to achieve particle size distribution similar to river sand, it can potentially function as fine aggregate in concrete mixtures. Owing to its exceptional properties, including low water absorption (0.6%-1.6%), dense microstructural framework, and high hardness, FNS demonstrates substantial potential as a fine aggregate in concrete applications. Extensive investigations have been conducted into utilizing FNS as a substitute for natural sand in concrete, with promising results reported across multiple studies. Saha and Chen [30, 31] demonstrated that incorporating FNS can enhance specific mechanical and durability properties of concrete compared to conventional formulations. Sun et al. [32] revealed that replacing fine aggregates with FNS significantly improves the chloride ion penetration resistance of concrete, particularly when combined with high proportions of ground blast furnace ferronickel slag (GBFS). Nguyen et al. [33] further indicated that FNS addition not only enhances the pozzolanic reactivity in the interfacial transition zone (ITZ) but also reduces water absorption, decreases permeable void volume, and improves resistance to chemically aggressive ion diffusion. Nuruzzaman et al. [34] reported that concrete containing up to 40% FNS fine aggregate complies with Self-Compacting Concrete recommended standards, exhibiting no segregation in flowability tests (J-ring method) and no blockage in passing ability tests (L-box and V-funnel methods). Liu et al. [35] observed notable improvements in sulfate resistance and abrasion resistance when FNS fine aggregate was introduced. Saha and Sarker [36–38] confirmed that FNS incorporation at replacement rates up to 50% effectively enhances concrete workability, compressive strength, and long-term durability, establishing its viability as a sustainable construction material.

Overall, FNS can be utilized as fine aggregate in concrete production, thereby reducing the consumption of natural sand. To further enhance the resource utilization rate of solid waste, this study aims to develop geopolymer concrete incorporating other mineral wastes. According to Salas et al. [39], geopolymer concrete exhibits a 64% lower global warming potential than conventional concrete in life cycle assessment studies. While replacing natural sand with ferronickel slag (FNS) demonstrates limited benefits in energy conservation and emission reduction, the feasibility of FNS-GC may offer a new pathway for reducing carbon emissions in construction materials. However, there is currently no research on the effect of nickel iron slag as fine aggregate on geopolymer nickel iron slag concrete. Therefore, this paper investigates the feasibility of FNS geopolymer concrete (FNS- GC). Specific tests include workability, compressive strength, and splitting tensile strength.

As an artificial composite material, the multi-component nature of concrete leads to significant dispersion in its mechanical properties, similarly, the variability in the performance of geopolymer concrete cannot be ignored [40]. This variability affects material stability and hinders its engineering application [41, 42]. Notably, the variability characteristics of FNS- GC strength remain unclear, especially when FNS replaces natural sand at different replacement ratios. In structural design, the primary concern regarding material dispersion lies in strength variability [43, 44]. Consequently, current codes universally adopt partial safety factors to ensure structural safety and reliability. Therefore, this research will focus on analyzing the statistical characteristics of strength in FNS- GC to elucidate its distribution patterns. The findings are expected to provide theoretical and experimental foundations for subsequent reliability studies.

## Experimental programs

### Raw materials

**Fig. 1:**
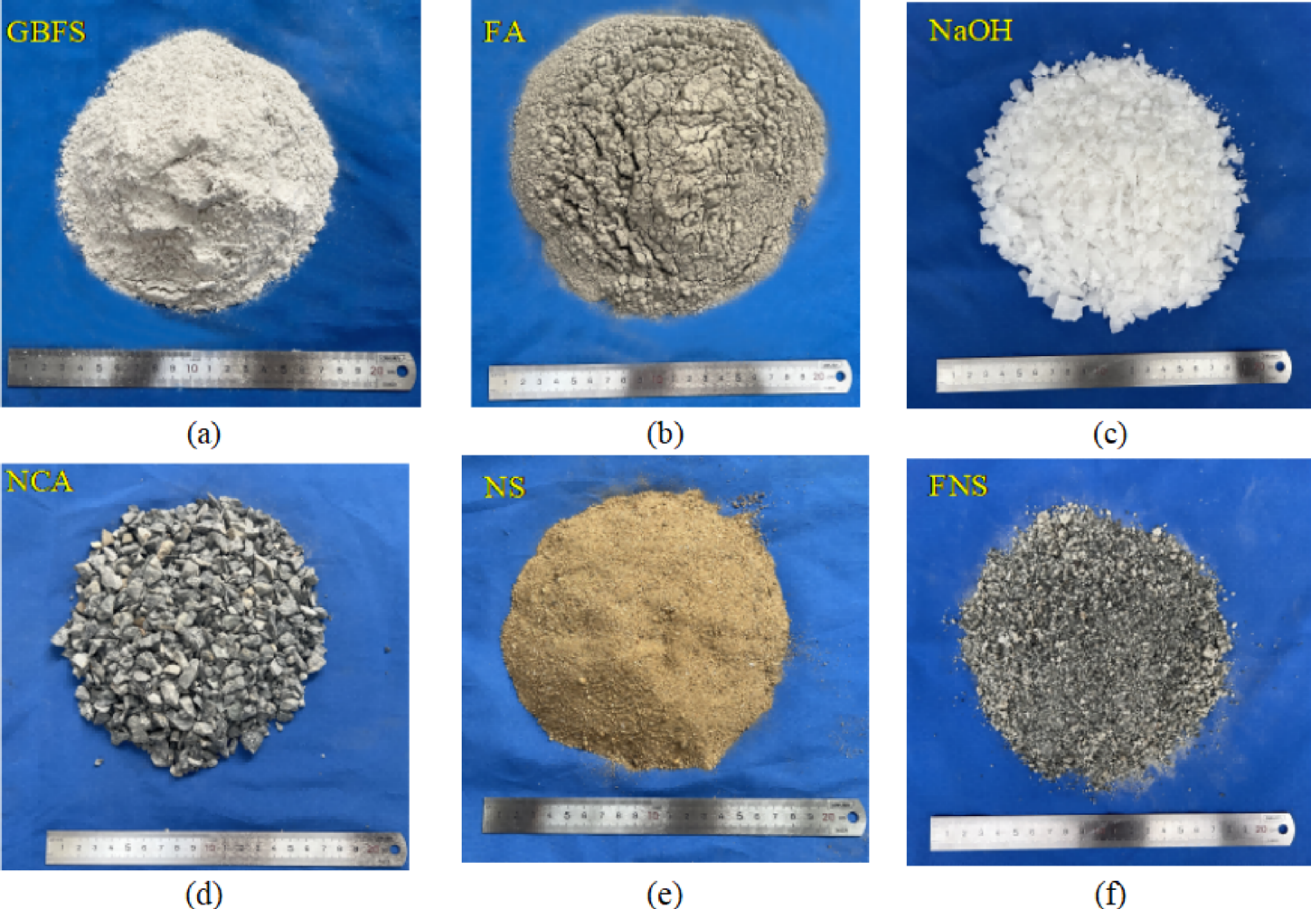
Raw materials: (**a**) GBFS; (**b**) FA; (**c**) NaOH; (**d**) NCA; (**e**) NS; (**f**) FNS.

**Fig. 2:**
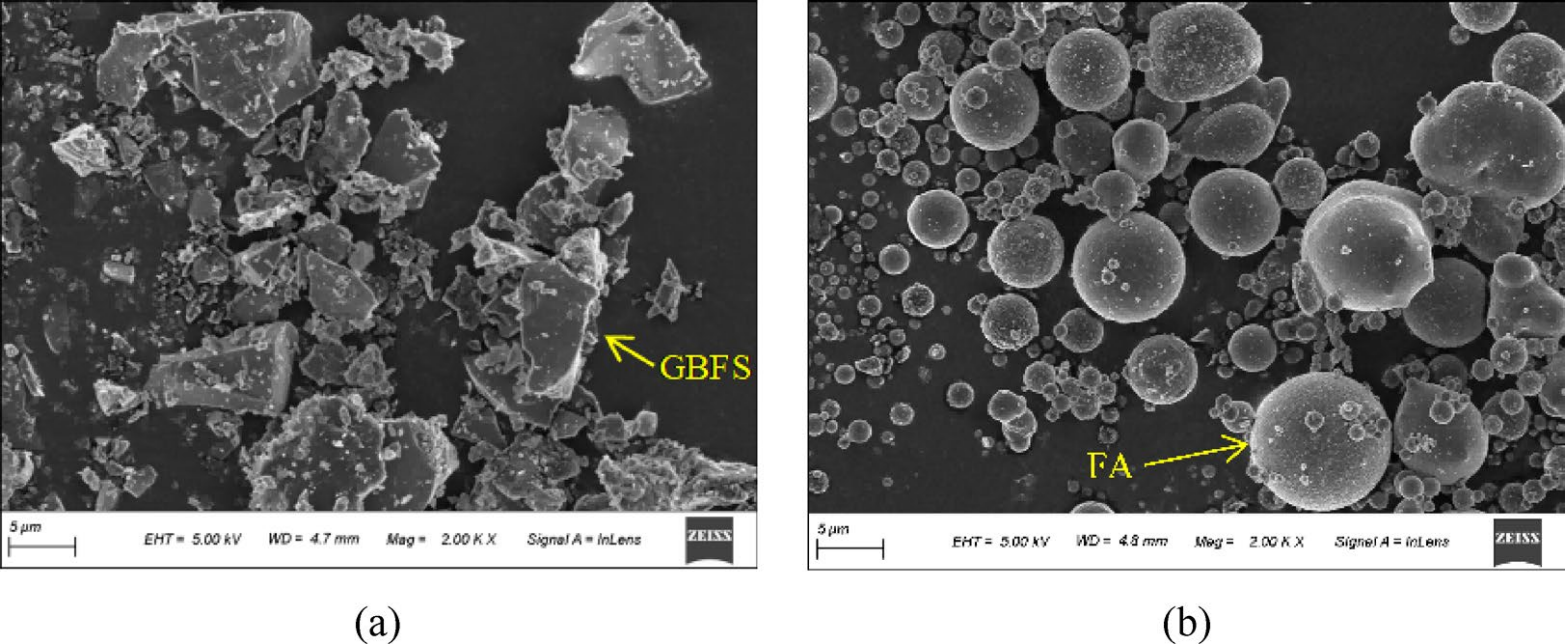
SEM results: (**a**) GBFS; (**b**) FA.

The materials used included S95-grade ground blast furnace slag (GBFS), Class F fly ash (FA), sodium hydroxide (NaOH), sodium silicate (SS), water, natural coarse aggregate (NCA), natural sand (NS), and ferronickel slag (FNS), as illustrated in Fig. 1. S95-grade GBFS and Class F FA were employed as precursor materials for geopolymer concrete. GBFS exhibited an off-white powdery appearance, while FA presented as a grey powder, both demonstrating excellent dispersibility. Laser diffraction analysis revealed the average particle sizes of GBFS and FA were 19.40 μm and 14.40 μm, respectively. Particle size significantly influences the activation reaction rate and strength development- generally, smaller particle sizes correlate with higher reactivity [12, 45]. Scanning electron microscopy (SEM) imaging of GBFS and FA (Fig. 2) showed that GBFS consisted of irregular crystalline structures with distinct edges and interfaces, whereas FA featured spherical particles of varying sizes, contrasting sharply with GBFS morphology.

Chemical composition is another critical factor affecting precursor activation. X-ray fluorescence (XRF) spectroscopy was used to characterize the chemical compositions of GBFS and FA, with results listed in Table 1. Both materials exhibited high contents of silicon and aluminum, but showed significant differences in calcium content.

**Table 1:** Chemical components of GBFS and FA (wt.%).

Oxide	SiO_2_	Al_2_O_3_	CaO	MgO	K_2_O	Fe_2_O_3_	Na_2_O	SO_3_
GBFS	33.43	14.93	38.86	7.25	0.47	0.38	0.29	2.45
FA	44.52	31.19	5.31	0.45	1.28	2.24	0.45	0.38

The solubility of SiO_2_ and Al_2_O_3_-key components of the precursors- exhibits exponential growth with increasing pH. OH⁻ ions catalyze the dissolution of Si^4^⁺ and Al^3^⁺ by hydrolyzing the Si–O-Si and Si–O-Al bonds in the precursors [45]. NaOH is widely recognized as an alkaline activator due to its ability to raise solution pH above 13.5 [45]. However, sole activation of aluminosilicate materials with NaOH often fails to achieve the target properties of the final product, necessitating the use of combined hydroxide-silicate activators [17, 46].

The activator was prepared by mixing liquid sodium silicate (SS) with solid NaOH. The target alkali modulus was 1.20 in this paper. The SS had an alkali modulus (Ms = mass ratio SiO_2_/Na₂O) of 3.2 and contained 27.3 wt% SiO_2_, 8.54 wt% Na_2_O, and 64.16 wt% H_2_O (content data provided by the manufacturer). The NaOH used was 99% pure solid flakes. The modulus (*Ms*) of the mixed alkali solution was calculated using Eq. (1) after adjusting the SS with NaOH additions.

1 $$m_{1} = \frac{{m_{ss} {\text{S}}_{{1}} }}{60}$$

2 $$m_{2} = \frac{{m_{{{\text{ss}}}} {\text{S}}_{{2}} }}{62} + \frac{{m_{{{\text{NaOH}}}} {\text{S}}_{{3}} }}{80}$$

3 $$M_{S} = \frac{{m_{1} }}{{m_{2} }}$$

where *S*_1_ represents the concentration (in %) of SiO_2_ in sodium silicate, *S*_2_ denotes the concentration (in %) of Na₂O in sodium silicate, *S*_3_ indicates the alkalinity (in %) of the alkaline solution;* m*₁ denotes the amount of substance of SiO_2_ (mol), *m*₂ represents the amount of substance of Na₂O (mol), *m*_ss_ is the mass of sodium silicate (g), *m*_NaOH_ is the mass of solid NaOH (g), and *M*_*s*_ signifies the target alkali modulus.

**Fig. 3:**
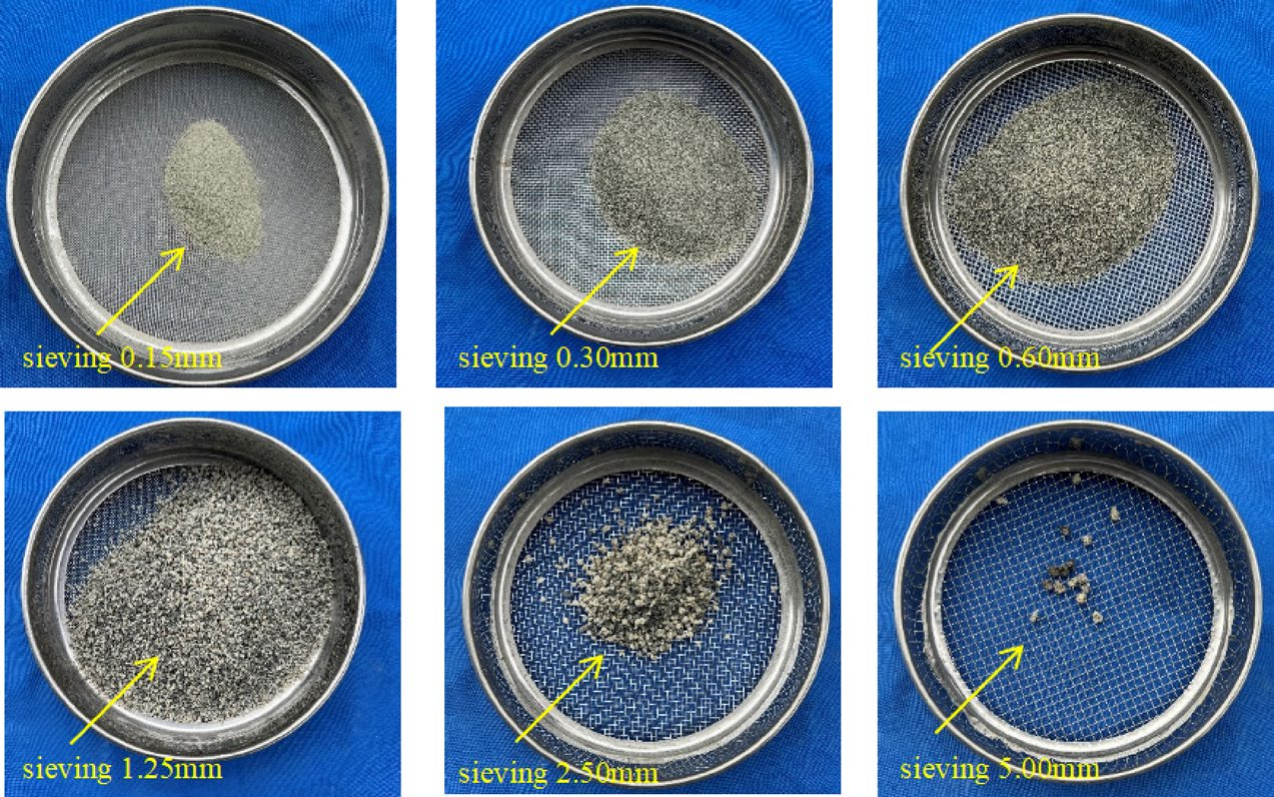
Different sieve size of FNS.

**Fig. 4:**
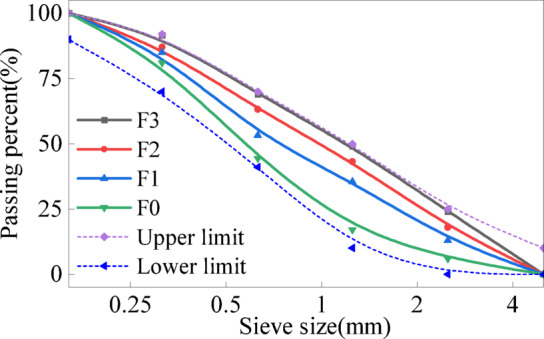
Particle size distribution of FNS.

The aggregates consisted of NCA, NS, and FNS, all sourced from Fujian Province, China. The NCA had a maximum particle size of 20 mm. The NS was river sand with a fineness modulus of 2.63 and a maximum particle size of 5 mm. FNS was derived from blast furnace ferronickel slag. In accordance with Chinese standard JGJ52-2006 [47], particle size distribution measurements were conducted on FNS. The results of the sieve analysis are shown in Fig. 3, and the particle size distribution of FNS is shown in Fig. 4, these indicating that FNS particles larger than 2.5 mm exhibit a porous and irregular morphology. As the FNS content increased, the fineness modulus of the fine aggregate gradually rose, classifying it as Zone II sand according to the standard.

The basic physical properties of NS and FNS were determined according to Chinese standard GB/T 14,684–2022 [48], with results summarized in Table 2. The data show significant similarities between NS and FNS. Notably, FNS exhibits a lower water absorption rate than NS, suggesting that increasing FNS content may increase free water content in concrete- a factor beneficial to workability. Additionally, the specific gravity of FNS is higher than that of NS, indicating that FNS-containing concrete has a greater density than NS-based concrete.

**Table 2:** Basic physical properties of fine aggregates.

Fine aggregates	NS	FNS
Fineness modulus	2.63	3.34
Water absorption (%)	1.87	0.94
Specific gravity	2.71	3.01

All aggregates were dried prior to use to ensure accurate moisture content measurement. To improve the fluidity of the mixture, a polycarboxylate-based superplasticizer with a water reduction efficiency of 25% was employed.

### Mix design and mixing process.

With the aggregate-to-binder ratio (A/B), water-to-binder ratio (W/B), and FNS replacement rate as influencing factors, a total of 16 mix proportions were designed. Specifically, the mass replacement rate of FNS considered were 0% (without FNS), 33%, 66%, and 100% (fully replaced by FNS). According to some research [49–51], the aggregate-to-binder ratio had three levels: 3, 4, and 5; and the water-to-binder ratio had three levels: 0.40, 0.45, and 0.50, as shown in Table 3. To accurately determine the statistical characteristics of the mechanical properties of FNS-GC, 18 cubic specimens with a side length of 150 mm were prepared for each mix proportion, and a total of three ages were considered, and for each age, three compressive strength test blocks and three splitting tensile strength test blocks were tested.

**Table 3:** Mix properties of concretes (kg/m^3^).

No	Specimen	FA	GBFS	Stone	NS	FNS	NaOH	Water glass	Water	Superplasticizer
1	AB3-WB0.45–0	153	358	920	614	0	27	139	134	8
2	AB3-WB0.45–33	153	358	920	411	203	27	139	134	8
3	AB3-WB0.45–66	153	358	920	203	411	27	139	134	8
4	AB3-WB0.45–100	153	358	920	0	614	27	139	134	8
5	AB5-WB0.45–0	107	249	1068	712	0	19	97	93	5
6	AB5-WB0.45–33	107	249	1068	477	235	19	97	93	5
7	AB5-WB0.45–66	107	249	1068	235	477	19	97	93	5
8	AB5-WB0.45–100	107	249	1068	0	712	19	97	93	5
9	AB4-WB0.4–0	127	296	1016	677	0	22	115	90	6
10	AB4-WB0.4–33	127	296	1016	454	223	22	115	90	6
11	AB4-WB0.4–66	127	296	1016	233	454	22	115	90	6
12	AB4-WB0.4–100	127	296	1016	0	677	22	115	90	6
13	AB4-WB0.5–0	125	291	998	665	0	22	113	130	6
14	AB4-WB0.5–33	125	291	998	446	219	22	113	130	6
15	AB4-WB0.5–66	125	291	998	219	446	22	113	130	6
16	AB4-WB0.5–100	125	291	998	0	665	22	113	130	6

A portion of the mixing water must be reserved for preparing the alkali activator. Prior to specimen fabrication, sodium silicate, sodium hydroxide, and the reserved water should be proportionally combined, vigorously agitated, and allowed to stand until cooled to ambient temperature. The pH of the activator must be measured at 1-h and 24-h intervals, with a permissible variation of less than 0.1 between measurements. Only the activator matured for 24 h is to be utilized in the preparation of geopolymer concrete test specimens.

**Fig. 5:**
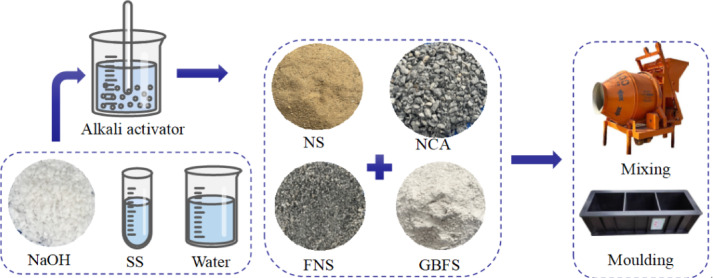
Specimen preparation diagram.

**Fig. 6:**
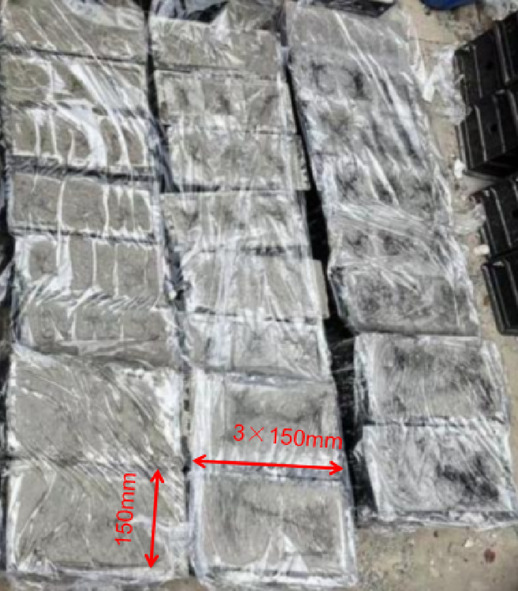
Preparation and maintenance of test specimens.

As depicted in Fig. 5, all dry constituents are blended according to predetermined ratios and mixed at low speed for 10 min to ensure homogeneity. Subsequently, water and alkali activator are introduced to the mixture, followed by 15 min of medium-speed mixing. During this stage, superplasticizer is incrementally added until achieving uniform consistency. Figure 6 illustrates the subsequent transfer of the mixture into molds using layered placement, followed by 2 min of mechanical vibration on a vibrating table to eliminate entrapped air. Specimens are immediately sealed with polyethylene film as a moisture barrier after vibration. Initial curing occurs at 20°C and 45% relative humidity for 24 h prior to demolding and specimen identification. Following demolding, specimens are placed in a standard curing chamber until reaching designated test ages (3-day, 14-day, and 28-day), with curing duration calculated from initial water addition.

### Test methods

#### Work ability

Under a specific water-binder ratio, the workability of concrete mainly depends on the properties of aggregates, such as gradation, particle size, particle density, and angularity. Well-graded aggregates can endow concrete with higher workability, and larger-sized aggregates require less water to achieve surface saturation [52]. Since workability directly affects the constructability of concrete, it is necessary to test the influence of FNS as fine aggregate on the workability of geopolymer concrete.

**Fig. 7:**
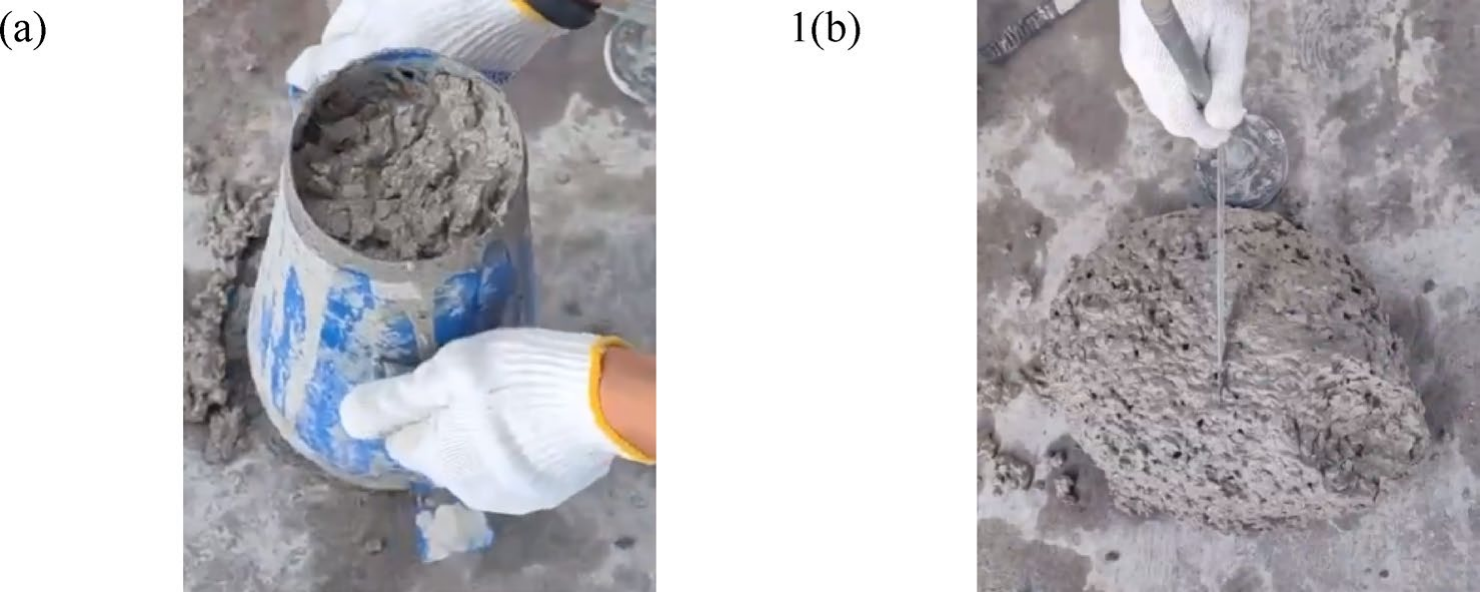
Test of slump.

The guideline GB/T 50,080- 2002 [53] specifies the use of the slump test to evaluate the workability of concrete, as shown in Fig. 7. By employing standardized tools, quantifiable procedures, and unified evaluation criteria, it transforms this simple method into a scientific and standardized process. This ensures that the test results remain unaffected by the operator’s subjective influence, thereby accurately, reliably, and comparably reflecting the workability of concrete. As a result, it serves as a critical basis for quality control and construction guidance of concrete.

#### Mechanical strength

**Fig. 8:**
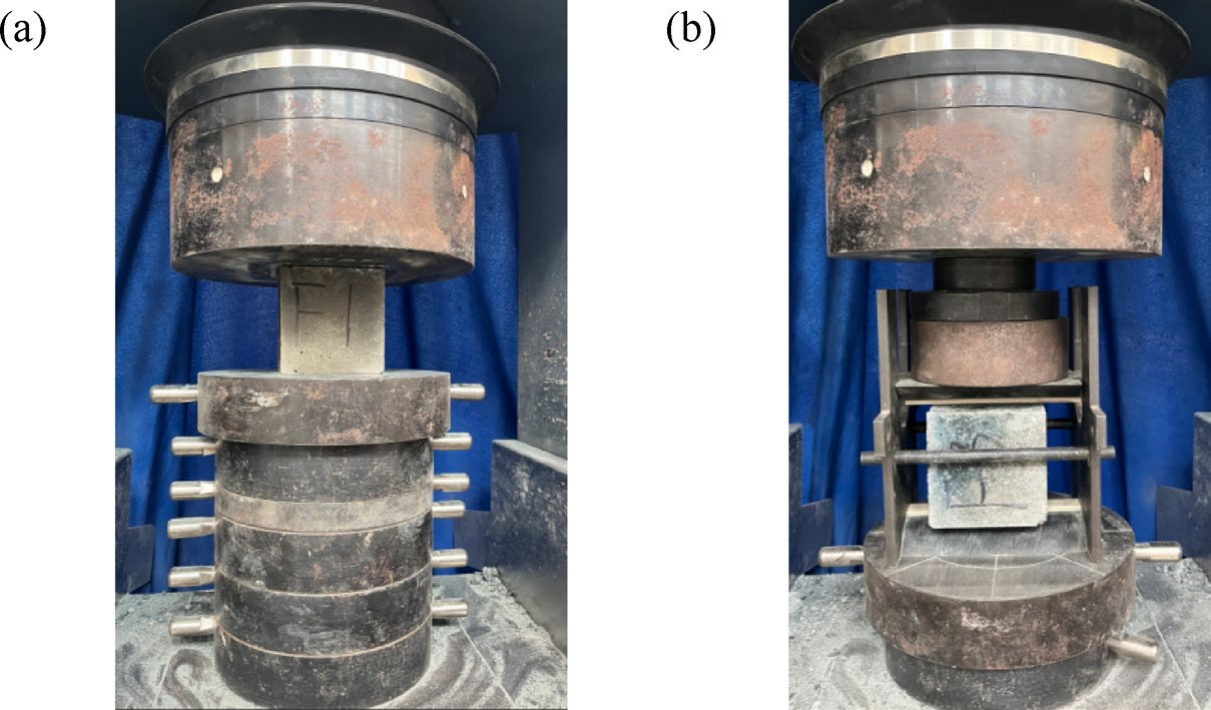
Test of strength: (**a**) Compressive strength; (**b**) Tensile strength.

To analyze the effect of FNS as fine aggregate on the strength of geopolymer concrete, compressive strength and splitting tensile strength tests were carried out on the specimens during the early hardening stage. Specimens with dimensions of 150 × 150 × 150 mm were taken out from the curing chamber at curing ages of 3 days, 14 days, and 28 days, respectively. After checking the integrity of the specimens and wiping their surfaces clean, the compressive strength and splitting tensile strength tests were conducted in accordance with the *Standard Test Methods for Mechanical Properties of Ordinary Concrete* (*GB/T 50,081–2019*) [54], and the calculated formula for compressive strength can be written as follows,

4 $$f_{cc} = \frac{F}{A}$$

5 $$f_{ts} = 0.637 \times \frac{F}{A}$$

where *f*_cc_ is the compressive strength of concrete cube specimen (MPa); *f*_*ts*_ is the splitting tensile strength of concrete (MPa); *F* is the failure load of the test piece (*n*); *A* is the bearing area (the splitting surface area of the test piece) (mm^2^) (Fig. 8).

#### SEM analysis

After the compressive strength test was completed on the specimens at the 28-day curing age, the test block samples from Group F1 were selected for scanning electron microscopy (SEM) analysis of slices. To observe these slices more clearly, they were immersed in absolute ethanol for 24 h to terminate the hydration reaction, then dried in a vacuum oven, and finally subjected to SEM analysis.

## Test Results

### Slump

**Fig. 9:**
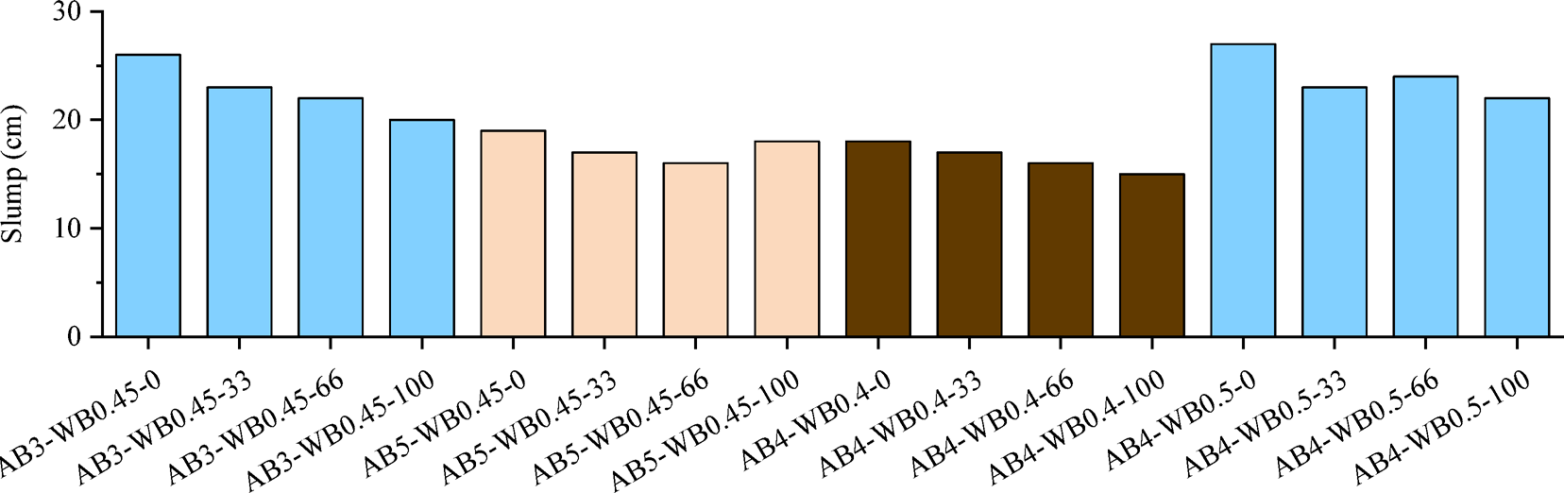
Slump of concrete.

Slump is a key indicator of workability, reflecting the fluidity and plasticity of the mixture. Figure 9 shows the slump results of various FNS-GC. Under the same water-binder ratio and aggregate-binder ratio, the higher the replacement rate of river sand (NS) by FNS, the smaller the slump of the concrete. For example, when the water-binder ratio is 0.45 and aggregate-binder ratio is 3, and when the replacement rate of FNS was 33%, 66% and 100%, the slump of concrete decreased by 11.58%, 15.38% and 23.08% compared with that without FNS.

The decrease in slump is due to the increase in the average particle size and the decrease in specific surface area of the mixed fine aggregate with higher FNS content, which reduces the coverage of cement paste on particle surfaces and decreases inter-particle friction. Additionally, FNS has more angular edges than NS, which greatly restricts the rolling of aggregates [36, 55]. Therefore, under the same water-binder ratio and aggregate-binder ratio, the fresh FNS-GC exhibits poorer workability.

The slump of the concrete is in the range of 16–26 cm. According to the guideline GB 50,164–2011 [56], mixtures with a slump value greater than 100 mm are classified as flowable concrete; those with slump values in the range of 50–100 mm are classified as plastic concrete; and those with slump values in the range of 10–50 mm are classified as low-plastic concrete. Overall, FNS-GC exhibits high fluidity, and this is similar to the other geopolymer concrete Refs. [57, 58].

### Strength

#### Compressive strength

**Fig. 10:**
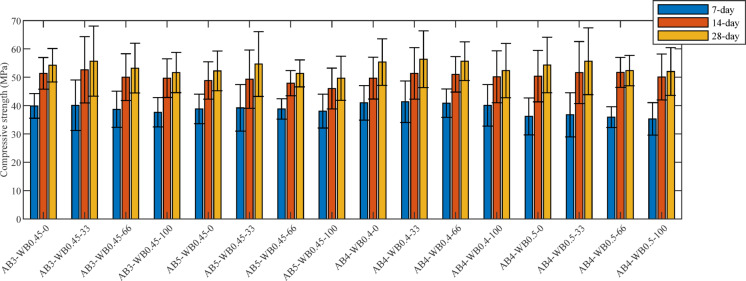
Compressive strength of FNS-GC.

Figure 10 presents the compressive strength of FNS-GC at different ages. The test results show that the FNS replacement rate exhibits a significant nonlinear influence on the compressive strength of FNS-GC, moreover, the use of FNS instead of NS will not significantly reduce the strength of concrete, and even improve the strength, which has also been confirmed in work of Bao et al. [52]. In general, the early strength age of concrete is high, and the compressive strength at 3 days and 14 days is about 72% and 94% of that at 28 days, respectively.

Under fixed aggregate-binder ratio and water-binder ratio conditions, a 33% FNS replacement rate generally enhances the compressive strength of concrete at all ages. For example, when the aggregate-binder ratio is 3 and the water-binder ratio is 0.45, the 28-day strength of the 33% replacement specimen (AB3-WB0.45–33) reaches 55.68 MPa, representing a 2.7% increase compared to the reference group (54.23 MPa). Similarly, in the group with an aggregate-binder ratio of 4 and a water-binder ratio of 0.4, the 28-day strength of the 33% replacement specimen (AB4-WB0.4–33) is 56.36 MPa, significantly higher than that of the reference group (55.35 MPa). However, when the replacement rate increases to 66% and above (e.g., AB3-WB0.45–100, AB5-WB0.45–100), the strength at all ages shows systematic degradation, indicating that excessive FNS may weaken matrix compactness due to imbalanced particle grading or increased weak interfacial zones. This strength evolution characteristic reveals an optimal replacement threshold for FNS (approximately 33%), where its micro-aggregate effect and active components can optimize the microstructure at appropriate dosages, but high contents will trigger negative effects.

Additionally, the synergistic effect between the aggregate-binder ratio and water-binder ratio significantly regulates the strength development path of FNS-GC. Lower aggregate-binder ratios (higher proportion of cementitious materials) are more conducive to strength improvement: at the same water-binder ratio (0.45) and 33% replacement rate, the 28-day strength of the aggregate-binder ratio 3 group (AB3-WB0.45–33, 55.68 MPa) was 1.03 MPa higher than that of the aggregate-binder ratio 5 group (AB5-WB0.45–33, 54.65 MPa), confirming that an increase in cementitious materials enhances matrix bonding force. Meanwhile, reducing the water-binder ratio significantly accelerates early strength development- when the aggregate-binder ratio was 4, the 3-day strength of the water-binder ratio 0.4 group (AB4-WB0.4 series) exceeded 40 MPa, while that of the water-binder ratio 0.5 group (AB4-WB0.5 series) was below 37 MPa. This phenomenon is attributed to the fact that a lower water-binder ratio promotes the polycondensation reaction rate of geopolymers, and this observation is consistent with the literature results [59, 60]. Notably, the combination of an aggregate-binder ratio of 4, a water-binder ratio of 0.4, and a 33% FNS replacement rate (AB4-WB0.4–33) achieved the highest 28-day strength (56.36 MPa), highlighting the significant synergistic effect among material proportioning parameters. This provides a key direction for proportion optimization in the resource utilization of FNS.

#### Tensile strength

**Fig. 11:**
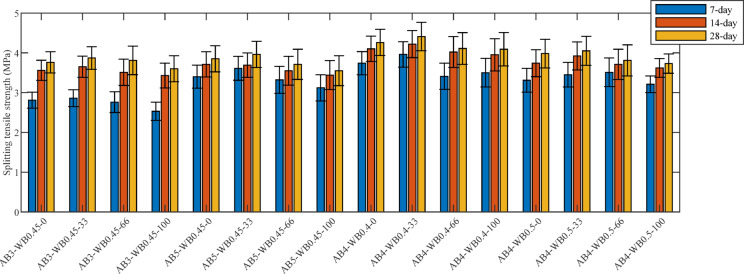
Splitting tensile strength of FNS-GC.

Figure 11 shows the splitting tensile strengths of FNS-GC at 3 days, 14 days, and 28 days. Similar to its compressive strength, FNS-GC demonstrates high early-age tensile strength, achieving approximately 84% and 95% of its 28-day split tensile strength at 3 and 14 days, respectively. The influence of concrete mix proportions on tensile strength exhibits no clear correlation pattern. Overall, mixtures with lower water-to-binder ratios demonstrate higher tensile strength. Notably, the split tensile strength of concrete displays a significant nonlinear trend with increasing FNS content- initially increasing then decreasing- reaching peak performance at 33% FNS replacement ratio.

**Fig. 12:**
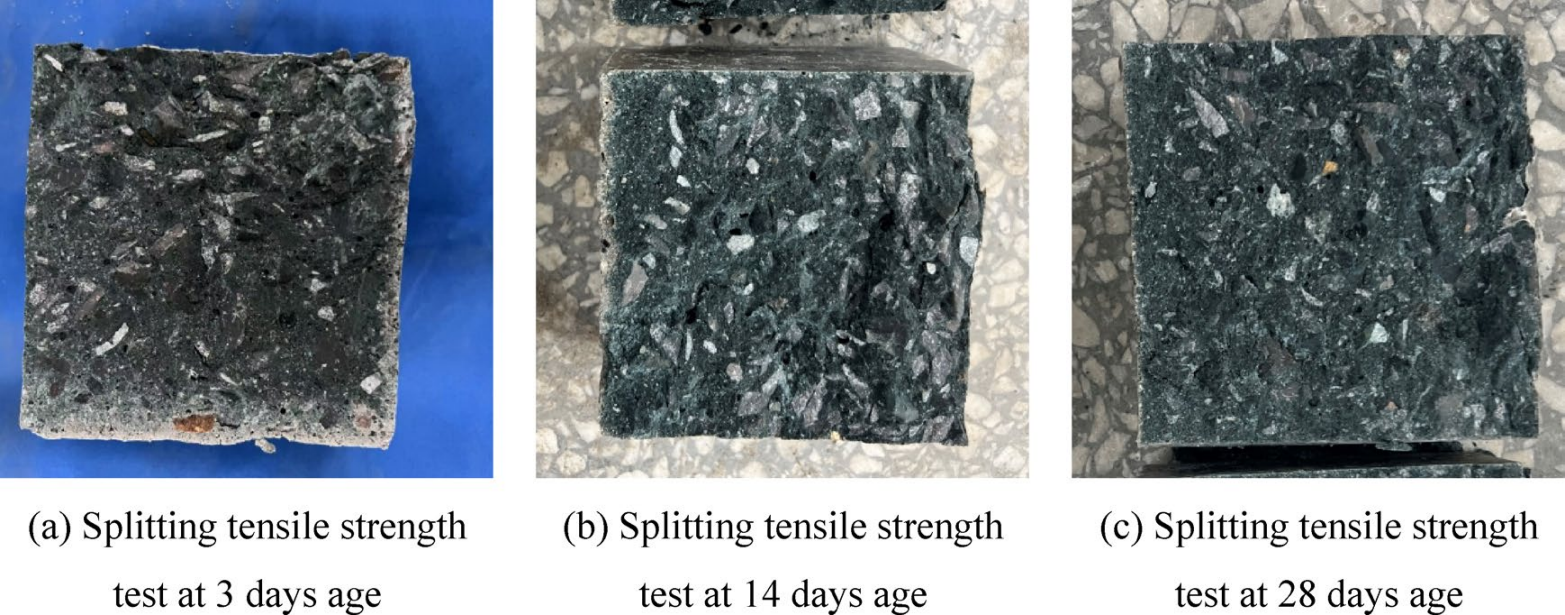
Concrete splitting surface.

Figure 12 shows the fracture surface of the concrete. It can be observed that with the increase in FNS content, the internal color of the specimen gradually deepens, accompanied by a reduction in the number of pores. These pores act as stress concentration points under loading, triggering crack propagation and weakening the matrix continuity, thus leading to strength degradation. This phenomenon confirms that the splitting tensile strength reaches its peak at a 33% FNS content and decreases as the content exceeds this threshold.

**Fig. 13:**
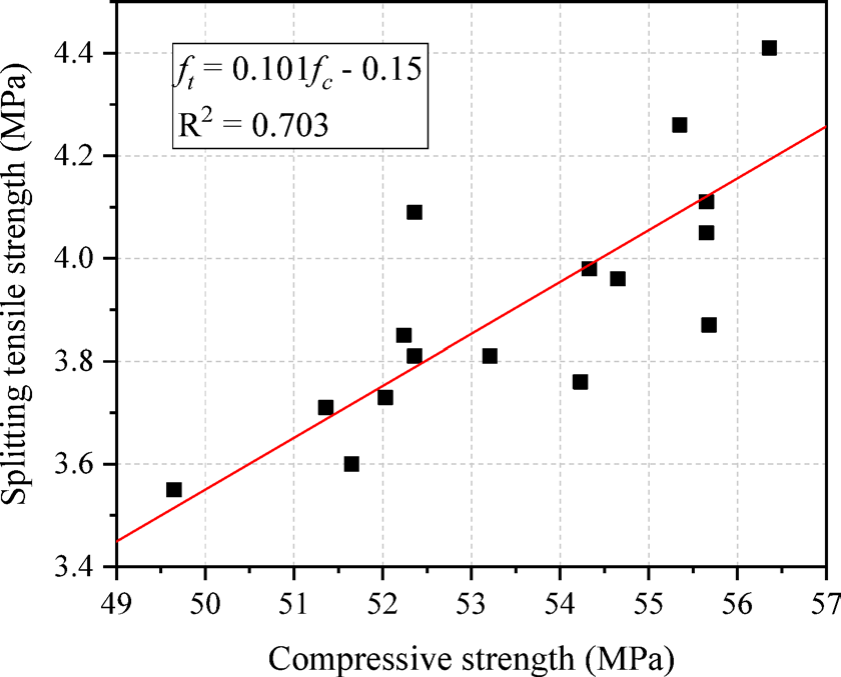
Comparison of splitting tensile strength and compressive strength.

In practical engineering, it is usually only necessary to test the compressive strength, while the splitting tensile strength can be derived from the compressive strength through conversion. Based on the measured tensile strength and compressive strength at 28 days, a prediction formula for the splitting tensile strength is obtained by fitting, as shown in Eq. (6). The comparison results and fitting results between the compressive strength and splitting tensile strength are presented in Fig. 13. Overall, the splitting tensile strength increases with the increase in compressive strength, so it can be approximately treated as a linear relationship. Compared with compressive strength, the variation law of splitting tensile strength with parameters is even less clear, which also leads to poor linear fitting effect between tensile strength and compressive strength.The coefficient of determination (R^2^) of the fitting is 0.703, and this value is lower than the R^2^ value of the linear fitting of other concretes [61]. In practical application, it can be handled flexibly according to the specific working conditions. If necessary, a split tensile strength test should be carried out.

6 $$f_{t} = 0.101f_{c} - 1.5$$

where *f*_*t*_ denotes the splitting tensile strength and *f*_*c*_ denotes the compressive strength.

### Scanning electron microscope analysis

**Fig. 14:**
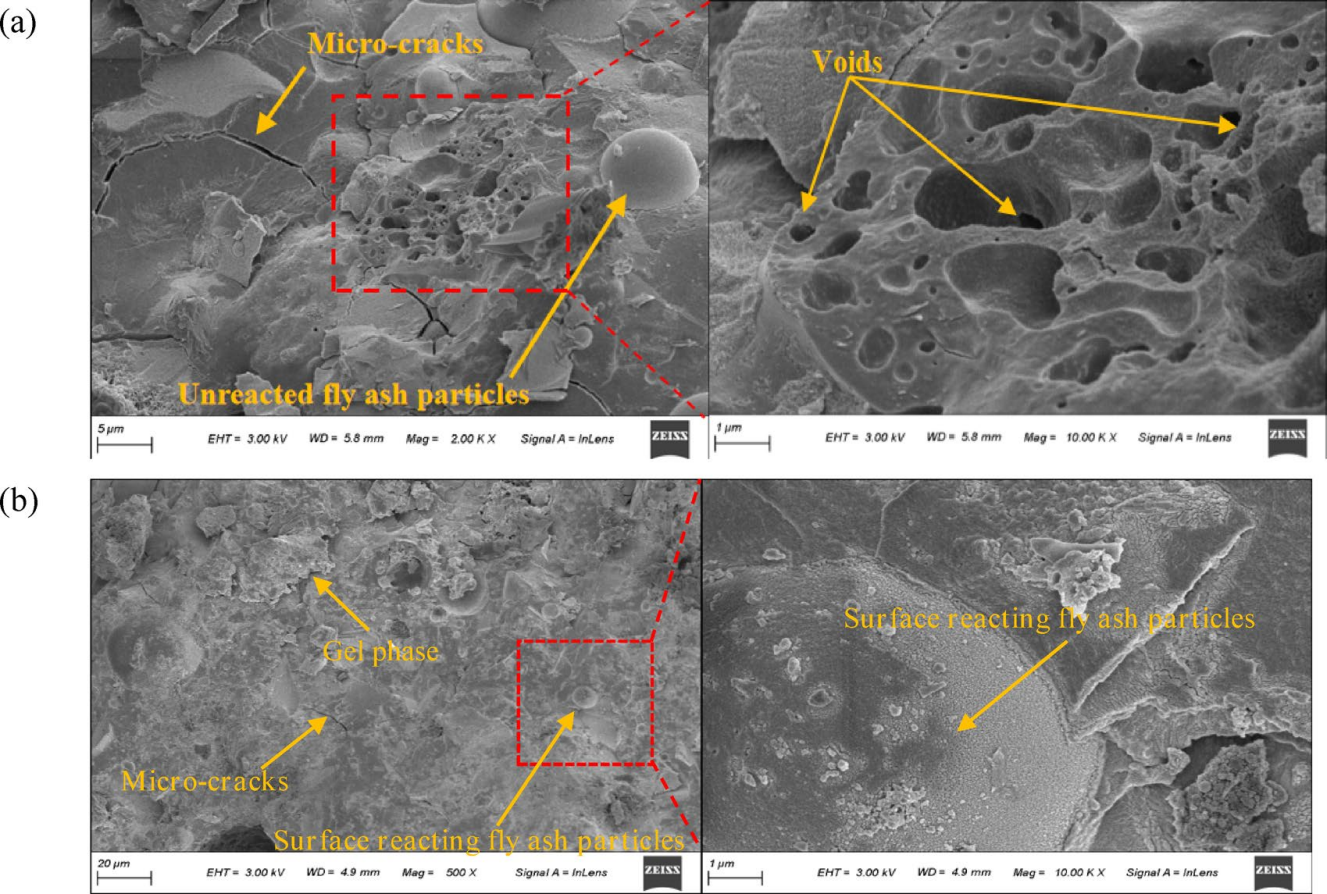
SEM results of FNS-GC.

Scanning electron microscopy (SEM) result of FNS-GC is shown in Fig. 14. Some FA remained unhydrolized, with its particles encapsulated by the cementitious phase and a smooth ITZ. The FA particles showed partial dissolution on their surfaces with a gel-phase appearance, while the particles were fully embedded in the gel matrix. This indicates that the reactivity of FA was reduced or inert under the alkaline activation of NaOH and SS, consistent with findings from previous studies [18, 62, 63]. Combining SEM results from other literature [32, 52], the intersection region between the cementitious phase and FNS fine aggregate exhibited a porous morphology, confirming that the rough texture of FNS provided stronger mechanical interlocking and adhesion at the matrix-aggregate interface.

## Discrepancy analysis

### Average values and coefficients of variation

**Fig. 15:**
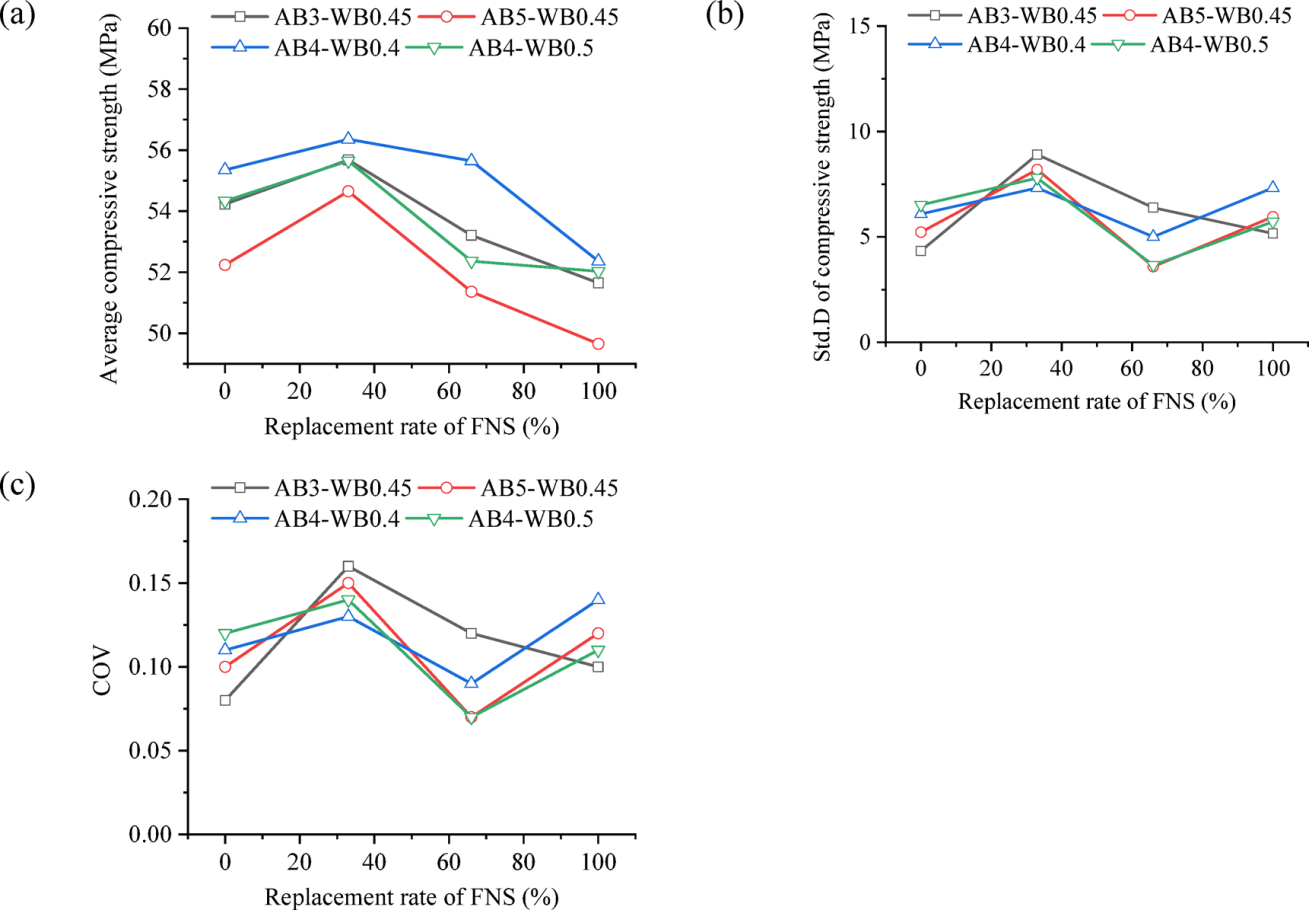
Discrepancy of compressive strength of concrete: (**a**) average compressive strength; (**b**) Std.D of compressive strength; (**c**) COV of compressive strength.

The average values and standard deviation value (Std. D) of the compressive strength of FNS-GC under different parameters are shown in Fig. 15. As observed, across various mix proportions, the mean compressive strength initially increases and then decreases with higher FNS replacement rates, peaking at a 33% replacement rate. The incorporation of FNS as fine aggregate in concrete leads to hydration reactions between the accompanying FNS powder, cement, and water, which to some extent enhances the interfacial strength between cement and FNS [64]. However, as the FNS content increases, the resulting improvement in interfacial strength is insufficient to compensate for the interfacial defects between FNS and the cement paste.

Mix proportion of FNS-GC shows no significant correlation with the standard deviation of compressive strength. The coefficient of variation (COV) for compressive strength ranges between 0.07 and 0.16 (see in Fig. 15c), and it is similar to the COV values reported for other types of concrete [65, 66]. The standard deviation of compressive strength initially increases and then decreases with rising FNS content, peaking at 33% FNS content where the COV reaches approximately 0.15. Notably, while the mean compressive strength achieves optimal performance at 33% FNS content, the increased COV reflects greater data dispersion. Consequently, practical applications must balance mean strength and COV to ensure structural design safety.

### Compressive strength distribution model

To investigate the distribution model of compressive strength of FNS-GC, the compressive strength of concrete with the same FNS replacement rate was normalized, and the specific expression is as follows,

7 $$f_{i} = \frac{{f_{c,i} }}{{\mu_{{f_{c} }} }}$$

where ${f}_{c,i}$ denotes the compressive strength of i-th test block; ${\mu }_{fc}$ denotes the average compressive strength of concrete with various mix proportion (excluding FNS replacement rate); ${f}_{i}$ denotes the normalized result of compressive strength of concrete.

After obtaining the normalized results of compressive strength of concrete with different FNS replacement rates, the distribution results of compressive strength were fitted using normal distribution, logarithmic normal distribution, and Weibull distribution. The expressions of these three distribution models are as follows:

8 $$f_{N} (\sigma ) = \frac{1}{{s_{1} \sqrt {2\pi } }}\exp \left[ { - \frac{1}{2}\left( {\frac{{\sigma - \mu_{1} }}{{s_{1} }}} \right)^{2} } \right]$$

9 $$f_{L} (\sigma ) = \frac{1}{{\sigma s_{2} \sqrt {2\pi } }}\exp \left[ { - \frac{1}{2}\left( {\frac{{\ln \sigma - \mu_{2} }}{{s_{2} }}} \right)^{2} } \right]$$

10 $$f_{W} (\sigma ) = \frac{k}{\lambda }\left( {\frac{\sigma }{\lambda }} \right)^{k - 1} \exp \left[ { - \left( {\frac{\sigma }{\lambda }} \right)^{k} } \right]$$

where *f*_N_(*σ*), *f*_L_(*σ*) and *f*_W_(*σ*) denote the probability density function of normal distribution, log-normal distribution , and Weibull distribution respectively; $\sigma$ denotes the compressive strength of concrete; *μ*_1_ and *s*_1_ denote the mean value and the standard deviation value of normal distribution; *μ*_2_ and *s*_2_ denote the mean value and the standard deviation value of log-normal distribution; *k* and *λ* denotes the shape and the scale parameters of the Weibull distribution.

**Fig. 16:**
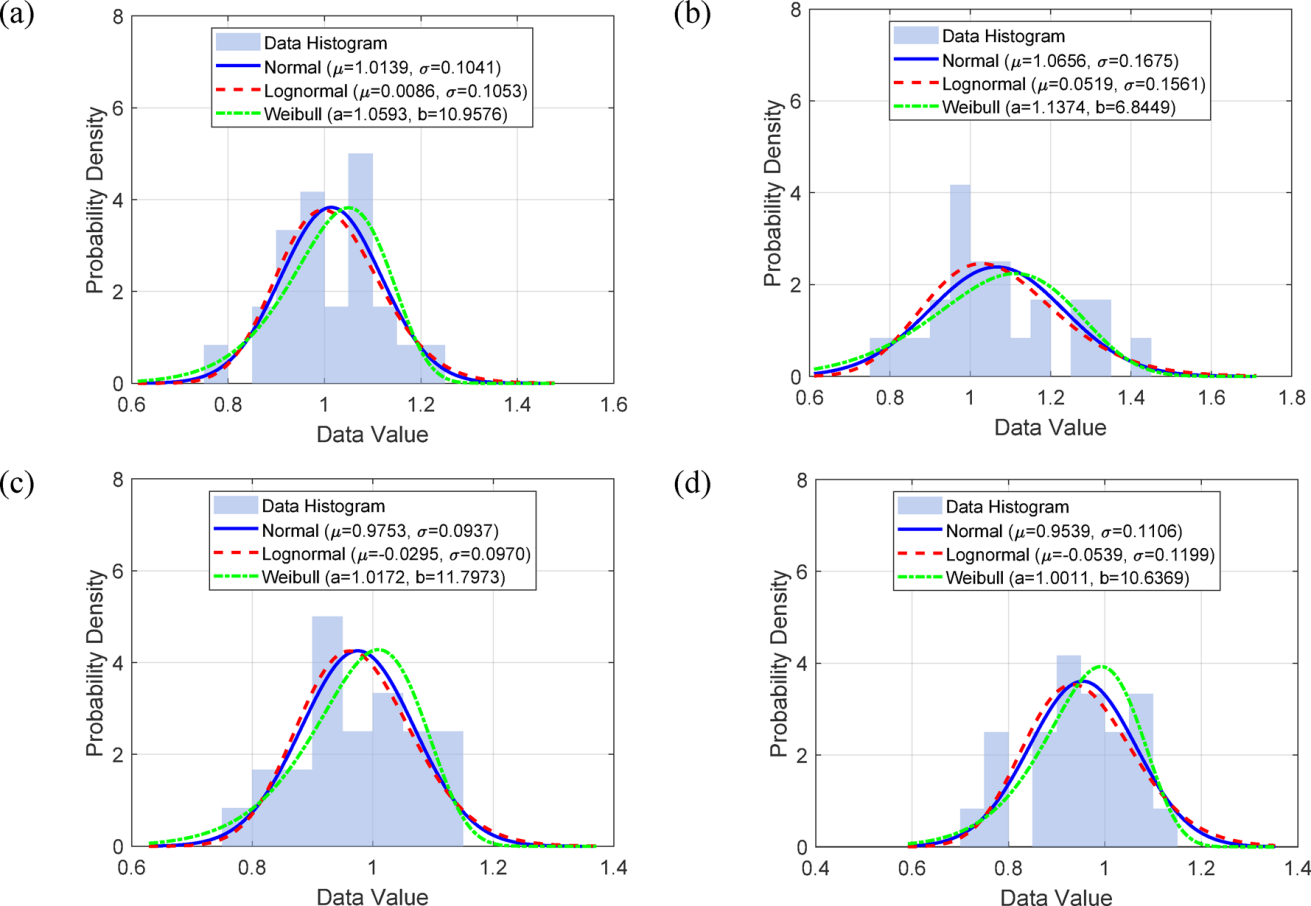
Distribution model of concrete compressive strength: (**a**) 0% replacement rate; (**b**) 33% replacement rate; (**c**) 66% replacement rate; (**d**) 100% replacement rate.

The distribution of normalized results of concrete compressive strength and the comparison of various distribution patterns are shown in Fig. 16. Overall, under different FNS contents, the normalized results of concrete compressive strength basically range from 0.7 to 1.3, exhibiting significant discreteness. The maximum degree of dispersion occurs when the FNS replacement rate is 33%.

The Kolmogorov–Smirnov hypothesis (K-S) test is a nonparametric method to compare distributions (one-sample vs. a model or two samples). It calculates the D-statistic (effect size), the maximum vertical distance between cumulative distribution functions (range: 0- 1). The *p*-value then estimates the probability of observing such an extreme D-value if the distributions were identical (null hypothesis). A small *p*-value rejects this hypothesis. The specific parameters and K-S test results of the normal distribution function, log-normal distribution function, and Weibull distribution model for concrete compressive strength under different FNS replacement rates are listed in Tables 4, 5, and 6. The results indicate that the fitting results of the three distributions do not reject the original hypothesis, among which the Weibull distribution model can more accurately reflect the statistical characteristics of concrete compressive strength, similar to that of ordinary concrete [67].

**Table 4:** Normal distribution coefficient of compressive strength of FNS-GC.

FNS replacement rate	*μ*_1_	*s*_1_	Effect size D	*p*-value	Result
0	1.0139	0.1041	0.097	0.961	Not reject
33%	1.0656	0.1675	0.130	0.763	Not reject
66%	0.9753	0.0937	0.068	1.000	Not reject
100%	0.9539	0.1106	0.121	0.834	Not reject

**Table 5:** Logarithmic normal distribution coefficient of compressive strength of FNS-GC.

FNS replacement rate	*μ*_2_	*s*_2_	Effect size	*p*-value	Result
0	0.0086	0.1053	0.113	0.888	Not reject
33%	0.0519	0.1561	0.103	0.938	Not reject
66%	0.0295	0.0970	0.078	0.996	Not reject
100%	0.0539	0.1199	0.144	0.647	Not reject

**Table 6:** Weibull distribution coefficient of compressive strength of FNS-GC.

FNS replacement rate	*k*	*λ*	Effect size	*p*-value	Result
0	1.0593	10.9576	0.126	0.799	Not reject
33%	1.1374	6.8449	0.157	0.543	Not reject
66%	1.0172	11.7973	0.108	0.916	Not reject
100%	1.0011	10.6369	0.091	0.978	Not reject

## Conclusions

To investigate the feasibility of utilizing ferronickel slag (FNS) as a replacement for river sand in geopolymer concrete (GC), this study designed and conducted experiments on GC mixtures considering different water-to-binder ratios, aggregate-to-binder ratios, and FNS replacement levels. The tested properties included workability (slump flow), compressive strength, and microstructure. Additionally, the statistical properties of the compressive strength were analyzed. The main findings are summarized as follows:

1. The slump of the tested concrete is 16–26 cm, which belongs to high fluidity concrete. The increase of replacement rate of FNS will reduce the slump of concrete. Increasing the aggregate-to-binder ratio or decreasing the water-to-binder ratio typically leads to reduced workability.
2. FNS-GC exhibits high early-age strength, with its 3-day and 14-day compressive strengths reaching approximately 72% and 94% of the standard 28-day strength, respectively. Overall, the compressive strength of concrete exhibits a significant nonlinear trend with increasing FNS content- initially rising then declining- reaching peak performance at 33% replacement ratio.
3. The splitting tensile strength of FNS-GC ranges between 3.2 and 4.4 MPa. It exhibits high early-age strength, reaching 84% and 95% of its 28-day splitting tensile strength at 3 days and 14 days, respectively. Similar to the compressive strength, the splitting tensile strength reaches its peak value at the 33% FNS replacement level. Overall, the tensile strength shows a positive correlation with the compressive strength, however, linear fitting models exhibit lower predictive accuracy for this concrete compared to conventional mixtures. Hence, the necessity of tensile performance testing should be evaluated based on site-specific requirements and structural criticality in engineering practice.
4. The compressive strength of FNS-GC exhibits considerable variability, with a coefficient of variation (COV) ranging from 0.07 to 0.17. The greatest variability was observed at the 33% FNS replacement ratio. The compressive strength distribution of FNS-GC is compatible with the normal distribution function, the log-normal distribution function, and the Weibull distribution function. Among these, the Weibull distribution provided the best fit for its distribution characteristics. In the subsequent research, this can be used as the basis to carry out the reliability analysis of the steel FNS-GC members, thereby obtaining the material partial factor of FNS-GC and providing a theoretical basis for the application of FNS-GC in engineering application.

Prior to the practical engineering application of any new concrete, its comprehensive performance characteristics must be thoroughly understood. While this study has focused on mechanical properties, future research should prioritize investigating long-term durability aspects such as sulfate/chloride resistance, shrinkage behavior, and freeze–thaw resistance.

## Supplementary Information

Below is the link to the electronic supplementary material.

Supplementary Material 1

## Data Availability

All data analysed during this study are included in this published article and its supplementary information files.

## List of symbols

FNS: Ferronickel slag
GC: Geopolymer concrete
FNS-GC: Ferronickel slag geopolymer concrete
GBFS: Ground blast furnace slag
GGBFS: Ground granulated blast-furnace slag
FA: Fly ash
NaOH: Sodium hydroxide
SS: Sodium silicate
SEM: Scanning electron microscopy
XRF: X-ray fluorescence
NCA: Natural Coarse Aggregate
NS: Natural Sand
A/B: Aggregate-to-binder ratio
W/B: Water-to-binder ratio
*f*_cc_: Compressive strength of concrete
*f*_*ts*_: Splitting tensile strength of concrete
*F*: Failure load of the test piece
ITZ: Interfacial transition zone
CV: Coefficients of variation
*f*_*c*__*,i*_: The compressive strength of i-th test block
${\mu }_{fc}$: The average compressive strength of concrete
${f}_{i}$: The normalized result of compressive strength of concrete
${f}_{N}\left(\sigma \right)$: Probability density function of normal distribution
${f}_{L}\left(\sigma \right)$: Probability density function of log-normal distribution
${f}_{W}\left(\sigma \right)$: Probability density function of Weibull distribution
K-S: Kolmogorov-Smirnov
Std. D: Standard deviation value
COV: Coefficient of variation
